# Cascading dominates large-scale disruptions in transport over complex networks

**DOI:** 10.1371/journal.pone.0246077

**Published:** 2021-01-25

**Authors:** Mark M. Dekker, Debabrata Panja

**Affiliations:** 1 Department of Information and Computing Sciences, Utrecht University, Utrecht, The Netherlands; 2 Centre for Complex Systems Studies, Utrecht University, Utrecht, The Netherlands; Central European University, HUNGARY

## Abstract

The core functionality of many socio-technical systems, such as supply chains, (inter)national trade and human mobility, concern transport over large geographically-spread complex networks. The dynamical intertwining of many heterogeneous operational elements, agents and locations are oft-cited generic factors to make these systems prone to large-scale disruptions: initially localised perturbations amplify and spread over the network, leading to a complete standstill of transport. Our level of understanding of such phenomena, let alone the ability to anticipate or predict their evolution in time, remains rudimentary. We approach the problem with a prime example: railways. Analysing spreading of train delays on the network by building a physical model, supported by data, reveals that the emergence of large-scale disruptions rests on the dynamic interdependencies among multiple ‘layers’ of operational elements (resources and services). The interdependencies provide pathways for the so-called *delay cascading* mechanism, which gets activated when, constrained by local unavailability of on-time resources, already-delayed ones are used to operate new services. Cascading locally amplifies delays, which in turn get transported over the network to give rise to new constraints elsewhere. This mechanism is a rich addition to some well-understood ones in, e.g., epidemiological spreading, or the spreading of rumours and opinions over (contact) networks, and stimulates rethinking spreading dynamics on complex networks. Having these concepts built into the model provides it with the ability to predict the evolution of large-scale disruptions in the railways up to 30-60 minutes up front. For transport systems, our work suggests that possible alleviation of constraints as well as a modular operational approach would arrest cascading, and therefore be effective measures against large-scale disruptions.

## 1 Introduction

Socio-technical systems such as supply chains, (inter)national trade and human mobility provide pivotal support to modern societies. Even though each one operates in its own intricate ways that are typically tuned to highly optimised benefit-to-cost ratios, their core functionality involves transport over geographically-spread, complex network backbones. Of particular interest are those situations in which—at detrimental costs to societies and economies—initial perturbations spread through a significant part of the network, leading to ‘large-scale disruptions’, i.e., near system-wide standstill of transport [1–3]. The initial perturbations are often triggered by exogenous events: indeed, well-documented are the world trade and supply chain disruption events that have been caused by natural disasters [4], and the ongoing COVID-19 pandemic [5]. Related research focuses on risks associated with critical infrastructures, service elements, alternative scenarios planning [6–10] and case studies of (potential) disasters [5, 11]. On the one hand, the dynamical intertwining of many heterogeneous operational elements and agents, network connectivity, and geographical spread are oft-cited generic factors responsible for the susceptibility of these systems to disruptions [12–16]. On the other, combined with a lack of reliable empirical data, the same factors also contribute to hinder the understanding, anticipation and prediction of systemic build-up of large-scale disruptions.

**Fig 1:**
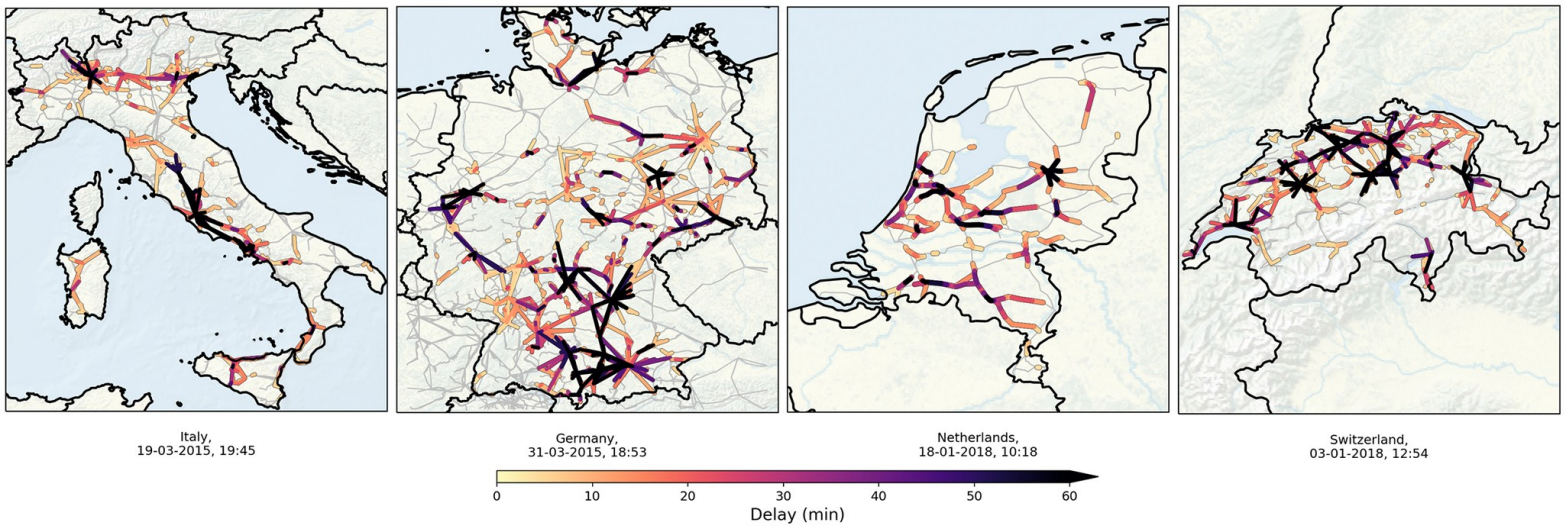
Examples of large-scale disruptions. Railway delays for strongly disrupted situations in four European countries (shown only are the delays larger than two minutes in colours; see SI section A for data description and sources). **Panel (a)**: near-simultaneous occurrence of several problems in the Italian railways in March 2015—a major one around Rome, affecting mostly intercity trains, and one between Milan and Venice. **Panel (b)**: effect of cyclone ‘Niklas’ (31 March 2015) on the German railways. In particular, a specific train near Pegnitz (center-south) was severely damaged by a fallen tree and the rooftop of the Munich station was destroyed, along with multiple smaller incidents across the country. The high risk of more accidents and delays caused the Deutsche Bahn to cancel most of its train activity throughout the day, leaving passengers stranded in major cities like Hannover, Frankfurt, Kassel and Berlin. **Panel (c)**: aftermath of storm ‘Friederike’ in January 2018 in the Netherlands, coinciding with an accident in the north of the country. Fallen trees and damaged overhead lines made the fire department force the Dutch railways to close at multiple stations—resulting in no train activity between the end of the morning and 14:00. A combination of the many disruptions with the lack of resources overview limited the possibility of mitigating delay at crucial corridors. The smaller scale and high density of the railway system in the Netherlands can be recognised also in Switzerland [**panel (d)**], where in January 2018 (coinciding with storm Burglind/Eleanor in the north-west of Europe) a strong disruption in near Zürich (north) rapidly propagated towards the rest of the country.

In order to address this problem, here we consider a prime example network transport system: railways. In most countries, railways witness large-scale disruptions—manifested by near system-wide train service delays—multiple times a year [13]. Large-scale disruptions in railways lead to heavy economic damage by hindering cargo and passenger transport [17]. Conversely, investments in robust railway systems may prompt economic growth [18, 19]. These especially hold true for densely urbanised countries in Europe, wherein railway transport, planned at a high density and frequency to match the extent of urbanisation, has developed to be an inextricable asset for societal and economic well-being, and is projected to be even more intensely developed due to rising demands and sustainability goals. While scattered delays are commonplace, they do occasionally build up to a large—near system-wide—scale, as illustrated for Italy, Germany, the Netherlands and Switzerland in Fig 1.

All cases in Fig 1, except Italy, were initiated by extreme weather: storms ‘Niklas’ (2015), ‘Friederike’ (2018) and ‘Burglind’ (2018, ‘Eleanor’ in the English nomenclature). *Exogenous* triggers like these—be it weather conditions, power outages [13], accidents or even earthquakes [17]—are typical for the onset of problems, but the consequences were driven by the system’s *internal* dynamics, propagating and amplifying delays to near system-wide scale. Indeed, in the year July 2017—June 2018, 29 days were marked as strongly disrupted days for the Dutch railways, with most of them occurring in November, December and January. This is no coincidence, even though railway companies use icing protection, adjusted timetables and many other precautions to prevent cold weather affecting their performance. Statistics like these, notwithstanding the wide variety of operations management for railway systems, highlight the *generic* aspects for the build-up of large-scale disruptions: while the initial *primary* delays, caused by external events, could possibly have been quickly resolved, systems’ internal issues cause new *secondary* delays, converting an initial locally-confined problematic event to an amplified near system-wide disruption.

For transport and logistic systems at large, considerable effort has been invested in identifying risks associated with criticality aspects of infrastructure in situations of hazards like in Fig 1 [9, 20] and how to deal with disruptions in terms of rescheduling [10, 12, 21]. Related literature aims to understand and predict the evolution of delays in transport systems, both under regular and disrupted circumstances. Most delay evolution models, however, focus on regular circumstances and predict how delay fluctuations develop using high-resolution statistics obtained from particular incidents or scenarios [20, 22], or from particular stations [23, 24] or lines [25–27]. These models come in various forms, mainly in the context of air and railway transport: analytical [28], agent-based [29], stochastic [30–33] and purely data-driven [3, 34]. A second relevant branch of transport literature focuses on robustness and vulnerability aspects, such as definitions of transportation resilience [35], perturbations in the network topology [9, 36], and data-based analyses on how the systems are connected [15, 37]. Specifically note that the above references include studies of multiple types of transport systems: airways, railways, supply chains, and even the analogy with freight truck and cargo ship transport. All these systems share the common feature of scheme-based transport, where disruptions may lead to subsequent delay of other transport units [38–41]. This effect, universal to many transport systems, is often commonly referred to as ‘cascading’ of delay; in this paper we analyse its contribution to large-scale disruptions for railways.

In the context of these models, which use the language of networks and dynamics, it is crucial to make the distinction between (a) dynamics *on* networks, where dynamics of a certain state variable evolve on top of, and is thus bound by (a time-invariant) network topology, and (b) dynamics *of* networks, i.e., involving (dynamic) links that can (re)appear, disappear or change weights (e.g., [9, 42–46]). In transport literature, the study of delay propagation generally conveys dynamics of the former type, while literature on infrastructure and resilience generally focuses on dynamics of the latter type.

Even though the above two paragraphs indicate transport literature as an active and broad field of study, there are still many unknowns, such as how the system as a whole evolves during disruptions, e.g., following an exogenous trigger. Due to heterogeneity in terms of space, time, human interactions and externalities that impact the system, the existing models typically lack accuracy and predictability (of evolution dynamics) in cases other than ‘regular’ (i.e., non-disrupted circumstances, or are purposed to simulate very specific scenarios, e.g., particular types of disruptions [47] or geographical areas [27, 48]. The contrast between disrupted and regular circumstances is manifold (and are visible in the results in this paper, e.g., Fig 4). Under regular, non-disrupted circumstances, delays are generally small and are of a less interactive nature with other delays, which allows for the applicability of data averages and a linear or local view [23]. The decreased interactivity of delays is a result of the fact that the schedules contain built-in buffers that, in case of delay, prevent the delay from affecting other transport units due to limited capacity of, e.g., platforms, tracks, or exchange of resources. Additionally, isolated delays can also be easily mitigated by human control. However, in case of (multiple) disruptions, the severity of delays exceeds the buffer and mitigation capacity and start building up and affecting other transport units, making the analysis rather complex [13]. For railways, Fig 1, provides a visual feel of delays building up and spreading across large spatial and temporal scales. The figure suggests that neither methods under regular circumstances, nor the specific incidents or scenarios covered in the existing literature are applicable to the underlying delay propagation mechanisms.

In this paper we focus on finding generic delay propagation mechanisms that do apply to situations as in Fig 1. Indeed, Fig 1 prompts us to conceptualise the intertwining of the many heterogeneous operational elements and agents in terms of stochastic processes playing out on a complex (infrastructure) network that remains invariant in time. We do so by constructing a structure with multiple layers of dynamics on a fixed infrastructure network—each layer of dynamics conveying the movements of a certain type of resource or service required for the system’s operations. This conceptualisation reveals that the interdependencies among the resources and services give rise to pathways for the delay cascading mechanism, which gets activated when, constrained by local unavailability of on-time resources, already-delayed ones are used to operate new services. Cascading amplifies delays locally for both resources and services, which in turn transport the amplified delays geographically over the network to give rise to new constraints elsewhere—describing phenomena we see in strongly disrupted situations like in Fig 1. Building the above concepts into a physical model leads us to reveal that the emergence of large-scale disruptions in networked transport requires three building blocks: constraints, cascading and transport. Not only does this paper bring new understanding of the evolution of disruptions, but the data-based interlinkages between transport resources and resulting cascading effect has also never been quantitatively shown in transport literature.

The model allows us to extract the key (delay-)amplifying role played by cascading, and to also predict the evolution of large-scale disruptions in the railways up to 30-60 minutes up front. We note here that the concept of cascading in itself is not new. Cascading in other dynamical systems like the Earth’s climate [49], or in network science in general is an active topic of research. On the one hand, in dynamics *of* networks, cascading has resulted in concepts like ‘cascading failure’, depicting the loss of connectivity in a network by a sequential removal of nodes and/or links [9, 43, 44, 50]. On the other, in dynamics *on* networks, such as innovation diffusion (e.g., in the work of Watts [51]) cascading refers to when an adoption, a rumour, an opinion or an infection process spreads through the entire network. In our work, the notion of cascading presented refers to interlayer spillover effects within the multiple layers of dynamics *on* a fixed infrastructure network, and it simply cannot be described by a diffusion-like model. Our work therefore stimulates us to rethink and contributes to broaden our horizon to spreading dynamics on complex networks (we will return to this discussion in Sec. 6).

The paper is structured as follows. In Sec. 2 we explain the cascading mechanism and how three ingredients may lead to large-scale disruptions: constraints, cascading and subsequent transport, and illustrate using a case example. In Sec. 3 we formulate a model from these building blocks. In Sec. 4, using the model, we quantify the role of cascading in driving large-scale disruptions. We investigate the performance and predictive power of the model in Sec. 5. We conclude the paper in Sec. 6 with a discussion on the broader outlook.

## 2 The three building blocks for large-scale disruptions

We start by pointing out the generalities of transport systems. They are based on a certain (infrastructure) network: e.g., rails and stations for railway transport, airports and airline services for air transport, and highways and cities for car transport. *Nodes* in a rail network correspond to stations between which *resources* (trains, personnel, scheduled lines) move on *edges*, which are the tracks between stations. In this paper, we treat the network as fixed or time-invariant, and study the system dynamics that is taking place on the network. The data for the Dutch railway system can be found in a repository on Open Science Framework (https://osf.io/tps4r/). Data of other national railway systems, used only in Fig 1, were found partially via another paper [52], i.e., from the following websites: OpenDataCity for German data (http://www.opendatacity.de/), ViaggiaTreno for the Italian data (http://www.viaggiatreno.it/) and OpenTransportData for the Swiss data (https://opentransportdata.swiss/). Details on time intervals and data considerations can be found in App. A.

As stated above, crucial to understanding the evolution of delay in case of a disruption is the impact and interactions of operational resources and agents. Using the example of railways networks, we conceptualise these elements by distinguishing three ‘layers’ of dynamics on the network, each of which has its own operator-specified scheme (i.e., dynamics) but is dependent on the schemes of the other layers: (a) a (train) service layer, containing the planned services, of which information is made publicly available via the timetable, (b) a rolling stock resource layer, involving the physical train units used to run the services, and (c) a crew resource layer, containing the personnel required to operate the trains. (Although we focus on railways, analogous formulation and dynamical layering is applicable to other transport systems.) For railways, resources and services couple by the condition that at least one rolling stock unit and at least two crew members (one driver and one conductor) are needed to run a service, leading to *dynamic couplings*—interdependencies—that link these layers along the (train) service routes. These interdependencies serve as the potential pathways for delay cascading in the following manner. Under regular circumstances, the layers act (largely) independently due to built-in local spare resource capacities and scheduled buffer times between service activities. However, new delays are generated when, constrained by local unavailability of on-time resources (i.e., when delays exceed buffer times and no spare resources are available), already-delayed ones are reused to run new services, activating ‘delay cascading’ (i.e., a process of delay generation at specific network nodes). Subsequently, these newly generated delays then get geographically transported along the service routes, and possibly create a similar constrained situation at some other node. (Most train services do not cross national borders, making cascading mainly a national problem, seen in Fig 1.) In this manner, constraints, cascading and subsequent transport of delays reinforce each other to make a localised delay perturbation amplify towards a large-scale disruption—this is exactly why we refer to these elements as the building blocks of large-scale disruptions.

**Fig 2:**
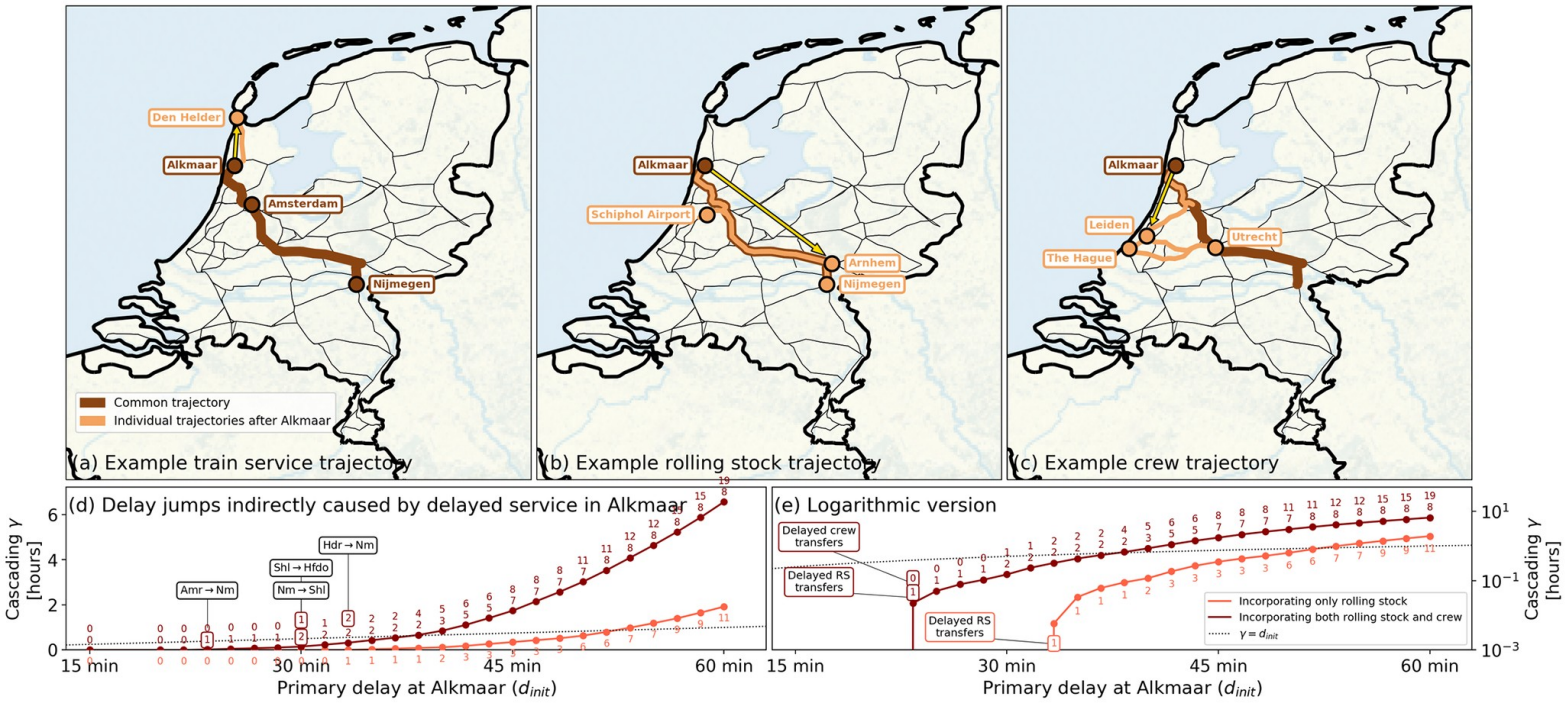
Illustration of delay cascading mechanism in the Dutch railways. **Panels (a)-(c)**: Routes in the Dutch railways of (a) the train service 3028 from Nijmegen to Alkmaar via Amsterdam, (b) a rolling stock unit used in part of this service and (c) a crew member (partly) executing this service. The schedules for 3 December 2017 are used, a day selected randomly from the dataset. Dark brown lines mark the route they share between Nijmegen and Alkmaar, after which they go their separate ways—marked in light brown lines and yellow arrows. While the service continues along its service route in panel (a) towards Den Helder, the rolling stock unit is coupled in Alkmaar onto another service to the south-east (leaving the service with only part of its original rolling stock)—via Schiphol Airport back to Nijmegen shown in panel (b). Panel (c) shows that the crew member transfers towards another service to the south-west—via Amsterdam to Leiden and The Hague, proceeding via Utrecht to Leiden and eventually ending in Utrecht. If service 3028 would have an initial delay *d*_init_ > 0, so does the rolling stock and crew executing the service; meaning that if scheduled buffer times for the resource transfers in Alkmaar would be exceeded by the delay (and no replacement resource would be available), then the subsequent services of these resources would become delayed as well. In other words, service 3028’s delay will potentially be transmitted to other services, and subsequently carried to other geographical regions. **Panel (d)** shows the *d*_init_-*γ* plot: *γ* remains zero for *d*_init_ < 23 minutes as the entire delay is absorbed by scheduled buffer times for resource transfers. However, with *d*_init_ = 23 minutes, a first resource delay overcomes the transfer buffer and adds delay to another service: namely the rolling stock unit in Alkmaar, going towards Nijmegen. As *d*_init_ grows, more and more transfer buffers are overcome and delay is added to many service lines throughout the country. **Panel (e)** contains the same information as in panel (d), for for a log-linear plot, revealing the near-exponential increase of *γ* as a function of *d*_init_. Abbreviations used in the panel depict Alkmaar (Amr), Nijmegen (Nm), Schiphol Airport (Shl), Hoofddorp shunting yard (Hdfo) and Den Helder (Hdr). The dotted black lines in panels (d-e) correspond to *γ* = *d*_init_; *γ* becomes larger than *d*_init_ for *d*_init_ ≥ 38 minutes.

Before we build these concepts into a physical model and quantify cascading, let us start with defining delay itself. All services and resources have planned and realised activity times; the planned ones constitute the *predefined scheme* of the railway operator. For a train service, every (discrete) activity *a*—in the forms of departures, arrivals or passings-by—can have a nonzero delay value *d*(*a*) = *t*_real_(*a*) − *t*_planned_(*a*), i.e., the activity is executed at a realised time *t*_real_ later [*d*(*a*) > 0] or earlier [*d*(*a*) < 0] than the planned time *t*_planned_. The change in delay of a train service’s activity *a* with respect to its previous activity is referred to as the *delay* *jump* *δ*(*a*). For example, consider a crew member coming from service X that needs to transfer in 10 minutes (i.e., the buffer time) to another service Y. Given that he is 16 minutes late from service X, the buffer time is exceeded and part of his delay from service X is transmitted to service Y if no replacement crew is available: it will cause a delay jump of 6 minutes for service Y’s next activity. Delay jumps in such situations can be suppressed by the use of spare resources or by buffers built in the schedule. Although affected by numerous factors, many large delay jumps are caused by delays cascading from one layer to another via resource-service interdependency [as illustrated in Fig 2(a)–2(c)].

## 3 The model

The model is built on the following premise: if a service line, rolling stock unit or crew member is delayed, then the delay is transported along the rest of its route, minus the buffers. We develop three variants of the model, all in the same vein of linking the full system’s resource schedules as discrete events: (1) the ‘monolayer model’, containing only the train services, wherein we explicitly incorporate the transport of delays along the service routes (it has no cascading), (2) the ‘bilayer model’, where we link the rolling stock layer to the monolayer model, and (3) the ‘trilayer model’, where we add the crew layer to the bilayer model. In the bi- and trilayer models delay also propagates *cumulatively* via rolling stock and crew: delay (minus built-in buffers) is passed on from one activity of a resource to the next activity performed by the same resource. The model we build has similarities to existing max-plus transport models [28], but is novel in (a) incorporating both rolling stock and crew layers, and (b) utilising real schedules for all resources at the full-system scale (see sections B and C for details in S1 File).

Following the prescribed scheme of the railway operator, the model is formally described as follows. Consider activity *a* of a certain train service line. Then consider the recent past activities {*a*′} of all resources (activities performed by the personnel, rolling stock units, and service line activities) used to execute *a* *prior* to *a*. Then, having denoted the delay for activity *a*′ by *d*(*a*′), the buffer between activities *a*′ and *a* by *β*(*a*′, *a*) and the Heavyside theta function by H [i.e., H(x)=1 if *x* > 0, and 0 otherwise], the model calculates *d*(*a*) as

$$\begin{array}{c} d\left(a\right)=\{\begin{array}{cc} \max_{\left\{a^{\prime }\right\}}\;\;\left\{H\left[d\left(a^{\prime }\right)-\beta \left(a^{\prime },a\right)\right]\right\} & \text{if}\;\left\{a^{\prime }\right\}\;\text{is}\;\text{non-empty}\\ 0 & \text{otherwise}. \end{array} \end{array}$$ (1)

In our related works, we have also experimented with an added noise term *ζ* to this model, allowing us to analyse the sensitivity of the results to noise. In general, it turns out that the cascading mechanism has a much stronger impact on the delay evolution than (Gaussian distributed) noise because cascading copies and amplifies existing delay rather than creating new (noisy) delay from scratch. In this paper however, we do not include the noise term, lest avoiding confusion between model noise and interpreted noise in the real data, as in Fig 4 (see also SI section C). Initialised at some time *t*_0_, i.e., being constrained to the train operator’s scheme predefined at *t*_0_, the model propagates initial delays to future (*t* > *t*_0_) ones via Eq (1).

For clarity, let us provide an example calculation of *d*(*a*). Imagine a train activity *a* that is ran by a train service *s*. Its previous activity *a*_prev_ had 30 seconds delay (no buffer), and we are interested in the delay of activity *a*. One of the rolling stock unit used for *a* came from elsewhere, where it had 300 seconds delay, with a buffer of 120 seconds (for recombining into service *S*). Two crew members transferred to service *S*, one (member *I*) with 720 seconds delay (with buffer 600) and one (member *II*) with 540 seconds delay (with buffer 600). Table 1 summarizes these numbers. The set {*a*′} in Eq 1 contains four activities, of which the buffers (third column) should be subtracted from their delays (second column), to get the potential contribution (fourth column) the delay of their combined activity, *d*(*a*). The corresponding delay jumps *δ*(*a*) are calculated by comparing the resulting *d*(*a*) for each model to the delay of the previous activity of the service *d*(*a*_prev_). In the monolayer model, we obtain *d*_mono_(*a*) = max{30} = 30 (such that *δ*_mono_(*a*) = 0), in the bilayer model *d*_bi_(*a* = max{30, 180} = 180 (such that *δ*_bi_(*a*) = 150) and in the trilayer model *d*_tri_(*a*) = max{30, 180, 120, 0} = 180 (such that *δ*_tri_(*a*) = 150).

**Table 1:** An example calculation of delay propagation in case of resource transfers. All values are stated in seconds.

Resource	Delay *d*	Buffer *β*	Potential contribution
Train service	30	0	30
Rolling stock	300	120	180
Crew member *I*	720	600	120
Crew member *II*	540	600	0

## 4 Quantification of cascading

In Fig 2(d)–2(e) we plot the *delay cascading metric γ*, defined as the cumulative sum of all resource transfer-related delay jumps, i.e., *γ* = ∑_*a*_ *δ*(*a*), consequential to some initial delay *d*_init_ due to planned service 3028 on 3 December 2017 at the city Alkmaar. Given a predefined buffer time scheme, the higher *d*_init_ is, the more buffers are exceeded; i.e., the more positive delay jumps occur, and the more *γ* increases. Stated differently, the ‘tighter’ the buffer times are planned—often the tendency of benefit-to-cost optimisations—the more prone transport and logistic systems would become to large-scale disruptions.

**Fig 3:**
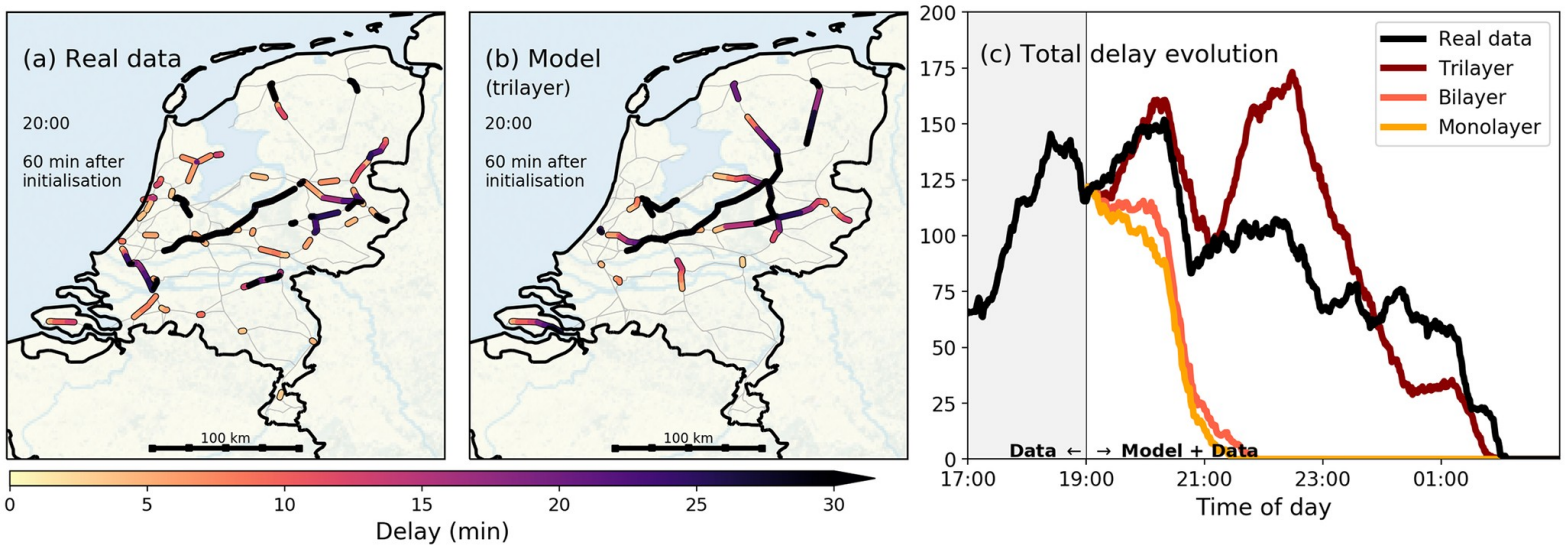
Model simulations of a real situation. Comparison of simulation and observed data on 11 December 2017. All models were initialised with the system snapshot at 19:00h. **Panels (a) and (b)** show the spatial distribution of delay at 20:00h in the real data (a) and simulation outcome of the trilayer model (b). **Panel (c)** shows the total delay evolution in time for the observed data and simulation outcomes of the three models. The trilayer model predicts the total delay well up to 120 minutes, after which it decays while in reality the total delay increased again. The differences between the monolayer model and the bi-/trilayer models stem from delay cascading, built in the latter ones. Only delays larger than 3 minutes are shown for visualisation purposes.

**Fig 4:**
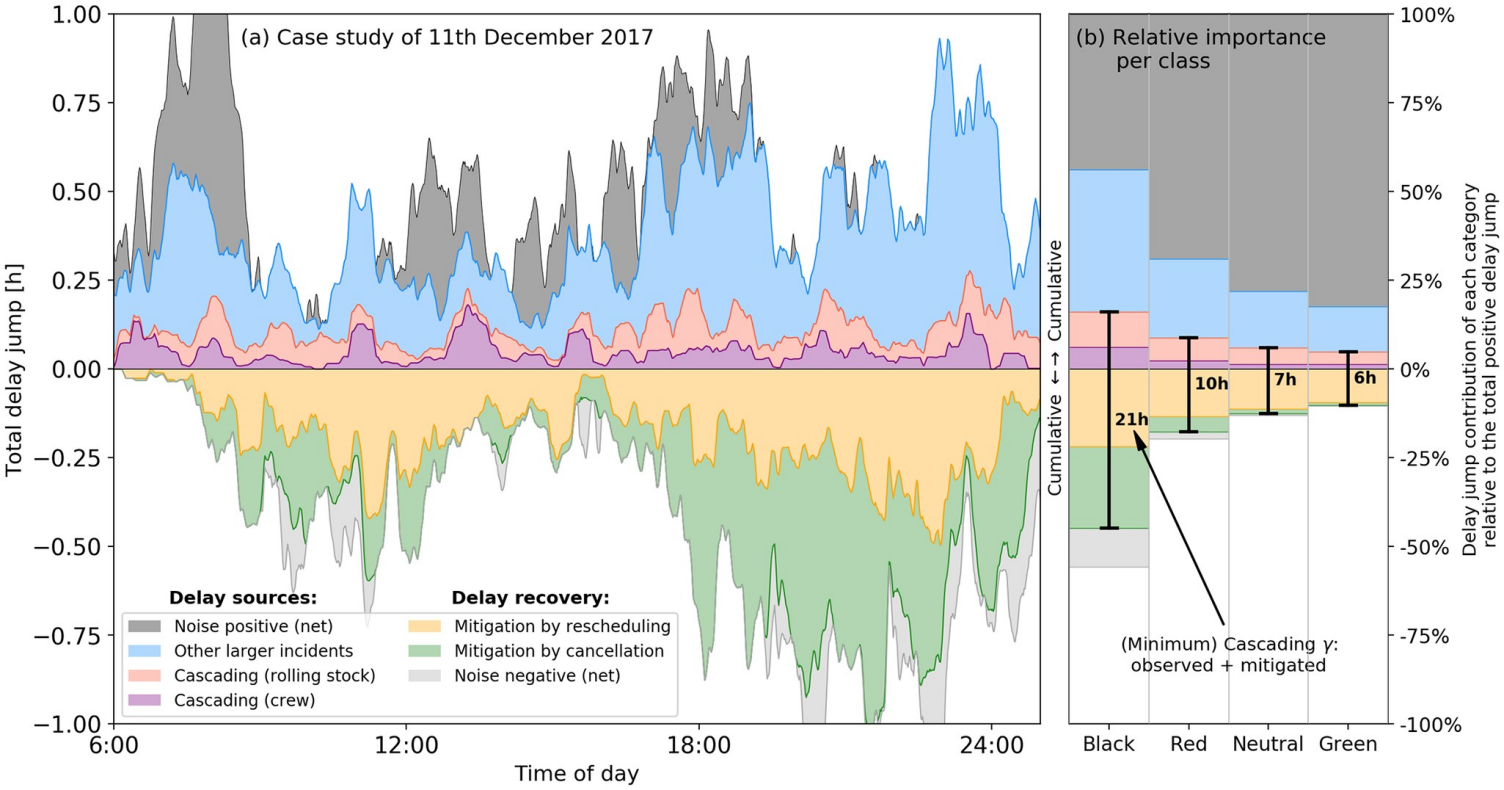
Origins of delay jumps. Total (summed) delay jumps, sorted by various mechanisms that cause them, and subdivided in ‘labels’, for **Panel (a)** one case study (11 December 2017), and **panel (b)** their proportions averaged over four days each for four day classes (‘Black’, ‘Red’, ‘Neutral’ and ‘Green’) for the Dutch railways. The origins of the delay jumps were identified by comparing observed data to model output. Magnitudes [panel (a)] and relative magnitudes [panel (b)] were calculated for time windows of 5 minutes, with a 30-minutes smoothening window used for display purposes. Four types of delay jumps that act as delay *sources* are distinguished: (I) delay cascading due to crew transfers (purple), (II) delay cascading due to rolling stock transfers (red), (V) other larger incidents (blue) and the positive part of (VI) net noise. Three types of delay jumps that act as delay *recovery* are distinguished: (III) mitigated cascading due to rescheduling (yellow), (IV) mitigated cascading due to cancellations (green), and the negative part of (VI) net noise. The positive part and the negative part are plotted separately in a cumulative sense—up- and downward, respectively. In panel (b), the total identified delay cascading (observed plus mitigated) is highlighted in hours.

In real-time operations, cascading like this will of course not go unnoticed, as railway dispatchers will take mitigation measures in real time. Rescheduling of rolling stock and crew, and service cancellations constitute mitigation measures; in effect, for *t* > *t*_0_, they simply alleviate (some of) the constraints imposed on the model by the operator’s scheme defined at *t*_0_. To briefly showcase how the model reproduces delay build-up in a real situation, let us now use it to evolve an actual delay snapshot of the *entire system*, and compare the results to the real data. To this end, we choose 11 December 2017 as a case study, another day with a severe blizzard (similar to the Dutch case shown in Fig 1). On this day, a code red weather alert was issued from 12:00h onward, in anticipation of which, the Dutch railways used an adapted schedule. Notwithstanding, the west and center of the country got disrupted to the point that almost no train traffic was possible around Utrecht and Amsterdam in the afternoon. Having initialised the model with the system snapshot at *t*_0_ = 19:00h—meaning also that the model is constrained to the operator’s predefined scheme at 19:00h—we compare the model predictions for the evolution of delay to the observed data at 20:00h in Fig 3. The aim of this figure is to illustrate whether our model reproduces amplification (in terms of delay magnitude) and geographic spread of delay well, when we initialise the model at a certain moment of time. Similarities between reality [panel (a)] and the model output [panel (b)] can be found in the center and east of the country, whereas differences can be seen in the south and north (such differences affect the model’s performance, analysed later). The total delays [panel (c)] for the mono- and bilayer models are seen to decrease quickly, while the trilayer model predicts the (increasing) evolution of the total delay rather well for the first 90-120 minutes. Note that the trilayer model prediction considerably overshoots the real data after 21:00h in panel (c). [In Sec. 4, we will demonstrate that the period 19:00-21:00 happens to coincide with a large number of mitigation measures (Fig 4a), suggesting that the dispatchers have mitigated much of the delay that the model predicts (this is consistent with the overestimation of the delay by the trilayer model)]. Still, the overall better performance of the trilayer model is logical, since in terms of modelling the entire system’s dynamics, this model is the most complete, and therefore it captures the fullest extent of the cascading effects. For this reason, we henceforth exclusively consider the trilayer model, referring to it simply as “the model”.

**Fig 5:**
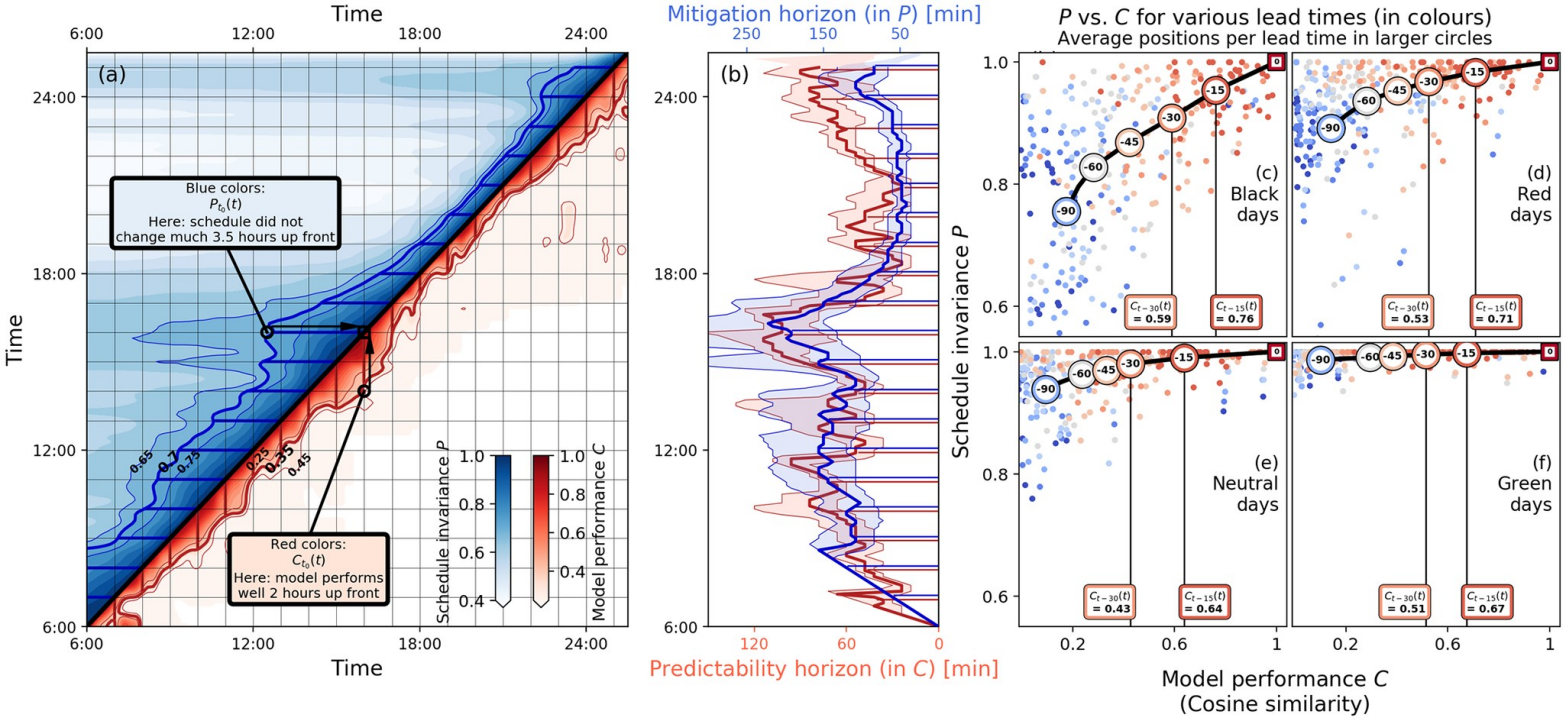
Predictability performance of the model. **Panel (a), above diagonal**: Mitigation measure $P_{t_{0}}\left(t\right)$ (blue), where both the model initialisation time *t*_0_ and crew activity time *t* are read from the horizontal axis. **Panel (a), below diagonal**: Model performance $C_{t_{0}}\left(t\right)$ (red), plotted similarly to $P_{t_{0}}\left(t\right)$, but times are read from the vertical axis. See also text for details. The contours $C_{t_{0}}\left(t\right)=0.35\pm 0.1$ and $P_{t_{0}}\left(t\right)=0.7\pm 0.05$ are marked respectively in blue and red lines. The data in this panel is smoothened using a Gaussian-averaging for visualisation purposes. **Panel (b)**: Horizons for *P* (blue) and *C* (red) for the same three values of *c* and *p* as in panel (a), measured as the horizontal and vertical distances to the diagonal of the *P* and *C* contours, respectively. **Panel (c)-(f)**: Model performance $C_{t_{0}}\left(t\right)$ (horizontal) versus crew schedule invariance $P_{t_{0}}\left(t\right)$ (vertical) on multiple instances of the day (*t* = 11:00h, 12:00h, …, 22:00h), with various lead times (*t*_0_ up to 1.5 hours before *t*), for the Green, Neutral, Red and Black days also analysed in Fig 4—16 days in total. Colours depict lead time: red indicates small lead times (i.e., predictions are closely up front), blue indicates large lead times. Averages on 15 min intervals are shown in large circles, with extra emphasis on the *C* values of these averages for 15 min and 30 min lead times (marked at bottom of each panel). Calculations of $C_{t_{0}}\left(t\right)$ and $P_{t_{0}}\left(t\right)$ are performed at *t*_0_ = 6-minutes time resolution [15 minutes for (c)-(f)], with a 30-minutes window around *t*.

In order to quantify the role of delay cascading in large-scale disruptions, we resort to a comparison of the delay jumps predicted by the model prediction and the operational data, as extracting this directly from the operational data is not possible since no cascading-related information, such as explanations for mitigation measures and cascading events, are logged. By initialising the model every 15 minutes between 06:00h on a day and 01:00h on the next, we first compute *all* model-predicted delay jumps with 15 minutes lead time. This lead time is chosen based on a high performance of the model (quantified below by *C* in Fig 5c–5f). We then heuristically classify them, upon comparing with operational data, in the following six categories: cascading due to (I) crew and (II) rolling stock transfers, mitigated cascading due to (III) rescheduling and (IV) cancellations, (V) other larger incidents (delay jumps > 10 minutes), and (VI) net noise (i.e., all other unaccounted for delay jumps), as follows (details in SI section C). If there was a positive delay jump at a rolling stock/crew transfer point in the operational data, and the model indicated a cascading event, then the delay jump is accordingly categorised as I or II. Other large and small delay jumps in observed data are categorised as V and VI respectively. Lastly, large delay jumps indicated by the model but not found in the operational data, upon cross-checking with the latter, are attributed to mitigation measures by rescheduling (III) or cancellations (IV). Note that category III concerns crew only; we lack real-time rolling stock rescheduling data since the Dutch railways infrastructure company ProRail does not record them. Further, the distinction between categories V and VI is artificial: it is primarily meant to demonstrate the occurrences of ‘large’ vs. ‘small’ incidents across day classes in Fig 4(b).

For clarification purposes, let us provide an example for the delay jump classification. Consider a train that departs from station A with 600 seconds delay, while earlier, upon arrival at this station (i.e., its previous logged activity), it had 180 seconds delay, resulting in an observed delay jump of *d*_obs_ = 600 − 180 = 420 seconds. With 15 minutes lead time, the model however happens to produce *d*_sim_ = 360 seconds delay. Since *d*_obs_ > 0 and *d*_sim_ > 0, we check the resource schemes, and find that the train re-departed from station A with a different crew than it had upon arrival at station A, and one of these crew members was delayed. We therefore mark the observed delay jump of 420 seconds as category I: cascading due to crew. Carrying out this procedure for all activities and associated delay jumps in the data, and adding their contribution together (per time window) results in the data that are shown in Fig 4.

Fig 4(a) shows the delay jump contribution of these categories for our case study day 11 December 2017 (note: this is the same day as in Fig 3); the respective contributions of these categories to the delay jumps can be seen to fluctuate heavily through the day—this is in fact typical for any day. In particular, many mitigation measures are found on this day, mostly in the evening, after 18:00h. By scaling up to four days each for four unique day-classes as used by the Dutch railways (Black, Red, Neutral and Green days), we show the relative contribution of each delay jump category in Fig 4(b). Quantitative details on this classification, can be found in SI section A. ‘Black’ days refer to days with many service cancellations and severe delays, while ‘Green’ days refer to days with barely any cancellation and only small delays (with ‘Red’ and ‘Neutral’ in between). For communication reasons we keep the label names as is used by railway practitioners.

Shown in approximate absolute hours Fig 4(b) are the aggregated contributions of each of the categories. When deriving the role of cascading from this figure, it is important to realise that categories I and II (cascading through crew and rolling stock) indicate a baseline portion of cascading: those that were detectable using the heuristic described two paragraphs above. The sizable magnitude of mitigation categories III and IV in Fig 4(a) indicates that many cascading-induced delay jumps were prevented by railway dispatchers. Further, categories V (‘other large incidents’) and VI (‘net noise’) cover a combination of externalities, coincidences, but also more cascading (which could not be captured by the heuristic) and secondary effects of cascading. It is for this reason that we identify the aggregated contributions of categories I-IV as the *minimum* (average) cascading metric *γ* for real-time operations. Fig 4(b) shows that *γ* decreases with the day-severity decreases: on Black days, we find an average of *γ* = 21 hours, while on Green days, this is only *γ* = 6 hours. The relation between *γ* and day-severity suggests that cascading indeed becomes increasingly important in more disrupted situations. (Also, the mitigation measures devised by the Dutch railways are evidently seen to be quite effective.).

## 5 Predicting the evolution of large-scale disruptions

Finally, we address the matter of predicting the evolution of large-scale disruptions using the model. How accurate the model predicts this evolution is referred to as the ‘model performance’. The relevance of model performance is twofold: for validating the model, and for providing (early) warnings to predict the evolution of disrupted situations in real-time operations. For the former, comparing model performance across multiple types of days (‘Green’, ‘Black’, etc.) may identify the cases wherein cascading, built in the model’s mechanisms (i.e., the three building blocks from Sec. 2), indeed played an dominant role in delay propagation (in cases when the performance is high), or when other (excluded) factors played a significant role (in cases when the performance is low).

In general, prediction by any delay propagation model suffers from two limiting factors: (i) large, poorly predictable, new incidents external to the system (captured partly by the blue and the gray areas in Fig 4), and (ii) mitigation measures (yellow and green areas in Fig 4) that alleviate constraints on the model imposed by the operator’s predefined scheme at initialisation time *t*_0_. By leaving these out by construction, our model outputs how delay would build up in their absence [this, in fact, is the qualitative explanation for the discrepancy between Fig 3(a) and 3(b), as remarked earlier]. The model’s prediction accuracy therefore gets limited to a certain lead time (*t* − *t*_0_)—the longer it is, the more negative influence (i-ii) have on its performance. We therefore define a *predictability horizon* (*t* − *t*_0_), for which the condition $C_{t_{0}}\left(t\right)=c$ holds for some *c*. Here $C_{t_{0}}\left(t\right)$ is the cosine of the angle *ϕ*, obtained from the dot product between the system-wide real and model-determined departure delay vectors $\vec{D}=\left[d\left(a_{1}\right),d\left(a_{2}\right),\ldots ,d\left(a_{n}\right)\right]$, obtained by aggregating all the train services in the time window *W*(*t*, Δ*t*) ≡ [*t* − Δ*t*/2, *t* + Δ*t*/2] with window size Δ*t* = 30 minutes:

$$\begin{array}{c} C_{t_{0}}\left(t\right)\equiv \cos\left[\phi_{t_{0}}\left(t\right)\right]=\frac{{\vec{D}}_{\text{real}}\left(t\right)\cdot {\vec{D}}_{\text{model},\;t_{0}}\left(t\right)}{|{\vec{D}}_{\text{real}}\left(t\right)||{\vec{D}}_{\text{model},\;t_{0}}\left(t\right)|}. \end{array}$$ (2)

We use this quantity as the *model performance metric*, with values between 0 (performing poorly) and 1 (performing perfectly). Although cosine similarity (like many other correlation metrics) does not incorporate absolute delay magnitudes when comparing model output to observed data since they are scaled out in Eq (2), the quantity *C* does take into account the spatial distribution as well as the corresponding relative magnitudes of delays (of which larger delays are of particular interest) over the entire network.

The lead time should in principle correlate well to the cosine similarity modulo the absence of the two limiting factors (i-ii). While we have little means to quantify (large external) incidents, we can quantify mitigation measures by $P_{t_{0}}\left(t\right)$, defined as the ratio (determined using the scheme at *t*_0_):

$$\begin{array}{c} P_{t_{0}}\left(t\right)=\frac{executed\;\text{crew}\;\text{actions}\;\text{scheduled}\;\text{to}\;\text{be}\;\text{in}\;W(t,\Delta t)}{all\;\text{crew}\;\text{actions}\;\text{scheduled}\;\text{to}\;\text{be}\;\text{in}\;W(t,\Delta t)}. \end{array}$$ (3)

(Ideally one should also account for rolling stock rescheduling in the interval [*t*_0_, *t*], but as pointed out earlier, we do not have data on live rescheduling of rolling stock.) Analogous to the predictability horizon, one can also define the *mitigation horizon* (*t* − *t*_0_) for which the condition $P_{t_{0}}\left(t\right)=p$ holds for some *p*.

Using the definitions (2-3), we first investigate the predictability horizon for our case study day 11 December 2017 (as in Figs 3 and 4a). Starting at 6:00h on the day, the model is initialised every 6 minutes, run forward, and both *C* and *P* are calculated at every time point (at 1 minute resolution) up to 01:00 AM on the next day for each run. The results are shown in Fig 5(a); both the horizontal and vertical axes display time of day. The diagonal represents *t* = *t*_0_, on which *C* = *P* = 1 trivially holds. Using the time-stamps displayed on the horizontal axis, in Fig 5(a) we plot $P_{t_{0}}\left(t\right)$ in blue at every point on the left of the diagonal—e.g., for the little black circle—where *t*_0_ is the model initialisation time-stamp of the point, and *t* is the crew activity time-stamp, corresponding to the intersection point of a horizontal line (shown by the right arrow) from that point and the diagonal. A similar process is followed for plotting $C_{t_{0}}\left(t\right)$ in red on the right of the diagonal, but this time using the time-stamps displayed on the vertical axis. The blue and the red contours for several values of *c* and *p* then respectively correspond to the mitigation and predictability horizons; they respectively measure how far ahead in time the crew schedule remains invariant enough, and how far up front in time the delay situation can be predicted. For example, the large distance of the *P* and *C* contours to the diagonal at *t* = 16:00h (following the arrows) means that the crew (that were working around 16:00h) were barely rescheduled prior to 16:00h, and the model also performed well to predict the delay state at 16:00h. Similarly, distance of the *P*-contour to the diagonal dropped considerably after 16:00 hour, resulting in large amounts of cascading mitigation, as in Fig 4a.

We expect that the two horizons to track each other for large-scale disruptions (the larger *P* is, the less the constraints at model start-time *t*_0_ are alleviated, meaning that the higher the model performance *C* ought to be). The relation between the two is explored in the *C*-*P* diagrams in Fig 5(b) and 5(c) in two different ways. The scatter of points in Fig 5(c) denote the *C* and *P* values for five different colour-coded lead times (*t* − *t*_0_) at hourly values of *t*, for the same four large-scale disruption (Black) days as in Fig 4(b). While there is a large scatter in Fig 5(c), their averages, plotted in circles marked with lead-time values, indicate a near-linear relation between *C* and *P* for lead time up to an hour. The same is seen in Fig 5(b), wherein the contour bands $C_{t_{0}}\left(t\right)=0.35\pm 0.1$ and $P_{t_{0}}\left(t\right)=0.7\pm 0.05$ from Fig 5(a) on 11 December 2017 are also seen to track each other well with an offset all through the day. Finally, Fig 5(d)–5(f) we compute the *C*-*P* diagrams for the other days as in Fig 4(b). For a given lead time, we find two distinct trends with increasing day-severity: (i) *P* decreases significantly [ostensibly to facilitate mitigation measures, as seen in Fig 4(b)], (ii) notwithstanding that, the model performance *C*—the cosine similarity—improves (in particular, the 30-min lead time averages improves from 0.43 and 0.51 on Green and Neutral days to 0.59 on Black days). This is indeed counterintuitive, but is explained by the following. The delay spreading mechanisms are better captured by the model on Black (severely disrupted) days in contrast to the Green days, for which delay spreading is dominated by incidents and noise events external to the model [covered by the blue and the gray areas in Fig 4(b)]. This corroborates our central message—namely that delay cascading dominates large-scale disruption events: despite more mitigation measures, more buffers are systemically exceeded on Black days than on, e.g., Green days.

## 6 Conclusion and outlook

To summarise: we show that large-scale delay evolution during railway disruptions emerge from the complex interactions of resources and services. Central to large-scale disruptions are the processes of delay amplification and spreading on the (fixed or time-invariant) infrastructure network. We find that these processes require a dynamical interplay among three building blocks: (a) constraints (the required resources are delayed), (b) cascading (this delay is passed on secondary services), and (c) transport (the secondary services spread the delay across long spatial and temporal time scales), wherein cascading playing the key role for delay amplification. Although we consider only railway systems here, the conceptual similarity in resource allocation and scheme-based dynamics in other systems like airways and logistic systems suggests that cascading can be an important driver to large-scale disruptions in these systems as well.

Although, in this paper we focus on railway transport with a wider outlook to transport and logistic systems at large, our work connects to a wider class of dynamical processes taking place on networks. In the introduction we have already pointed out that the problem we study here—spreading phenomena on fixed (time-invariant) railway infrastructure network—belongs to the type of dynamics *on* networks. Well-studied examples of this type are diffusion-related phenomena on networks (e.g., in various (bio)chemical reaction-diffusion, or activation-inhibition processes running on networks [53, 54]), network epidemiology [55], and the spreading of rumours and opinions over social networks [51, 56]. Even though one can make a distinction among these regarding nature-made or human-made systems subtypes, the spreading mechanism we identify here, *viz*., delay cascading across multiple layers of resources and service elements, is a rich (and supported by real life data) addition to literature of dynamics on networks. The constraint-cascading-transport mechanism of (delay) spreading, which we derive here using the example of railway transport, adds to the understanding of the spreading dynamics on complex networks. While we hope that our work will stimulate a wider search into spreading phenomena on complex networks, we also note that the multi-layer coupled dynamics can also be formulated as a temporal network [57], which is however beyond the scope of the the current work.

Finally, for the specialist field of transport research, we foresee two effective measures to arrest cascading, potentially averting large-scale disruptions. First, the introduction of ample spare resource capacities and buffer times, to ensure that local constraints do not get easily activated. The trilayer model pinpoints precisely where vulnerabilities lie within the railway operator’s scheme: e.g., in Fig 2 we show that delays in the Dutch city of Alkmaar (north-west) at a particular moment in time has the potential to cause delays in the city of Nijmegen (east) at a later point in time: a form of building long-range causal correlations in the system. Spare crew in Alkmaar would not only prevent this specific spread, but upscaling the model’s results to long-term statistics would also provide any transport system’s dispatcher with data to pinpoint ideal locations and quantities of spare resources—an analysis we plan to execute in the future. (Having said this, we acknowledge that optimising the system for benefit-to-cost ratios will limit the possibilities of having spare resources.) A second measure to prevent cascading is a modular design approach for the transport functionalities. Modular designs (like the Danish railway system), by definition, do not reuse resources from one area to another, which prevents delay cascading between these regions. Modular design approaches have been considered in the context of operational rescheduling during disruptions [21, 58]. We hope that our paper prompts new research into the trade-offs and complexity of how to design transport systems resilient to disruption spreading.

## Supporting information

S1 File (PDF)
