# Supplementary material for: Cascading dominates large-scale disruptions in transport over complex networks
Source: PLoS One. 2021 Jan 25;16(1):e0246077. doi: 10.1371/journal.pone.0246077 (PMC7833156; doi:10.1371/journal.pone.0246077)
Supplement: S1 File — (PDF) [file pone.0246077.s001.pdf]

Supporting Information - *Cascading dominates large-scale disruptions in transport over complex networks*

Mark M. Dekker[1,2*], Debabrata Panja[1,2]

1 Department of Information and Computing Sciences, Utrecht University, Utrecht, The Netherlands
2 Centre for Complex Systems Studies, Utrecht University, Utrecht, The Netherlands

* m.m.dekker@uu.nl

A Data

A.1 Data source for the Dutch railways

Realised data (i.e., delay information) and train line schedules were obtained from ProRail, and rolling stock and crew schedules were obtained from the main Dutch railway company Nederlandse Spoorwegen (NS). Although the latter is not a complete set of all rolling stock and crew schedules, NS covers about 90% of all train activities in the Netherlands, allowing for relative completeness in most of the country. All assets are anonymised for privacy reasons and can be tracked within each individual day. For the rolling stock schedules on any given day, only the realised schedules are recorded (due to the nature of how these assets are planned), while for the crew data, both realised and planned schedules are fully known, including all intermediate mutations to the schedule. We focus on passenger trains only, and exclude freight trains in the analysis. The main reason for this is that freight trains are (economically) privacy sensitive, meaning that it is difficult to get a complete dataset. It should be noted that their contribution to delay is usually only minor, as they cover only 5.7% of all Dutch train kilometers in 2017 (numbers courtesy of ProRail), and having schedules and routes that are often partly separated from the passenger train dynamics. More details can be found in Dekker et al. (2019) [1], section 3.1. Results in this paper are obtained from a dataset ranging from 1 July 2017 to 30 June 2018. The Dutch data can be found in an Open Science Framework repository (`https://osf.io/tps4r/`).

A.2 Data source for the other railway systems

Data for the German and Italian railways in Fig. 1 is gained from the supplementary material of [2], which got this data from the OpenDataCity (`http://www.opendatacity.de/`) and the ViaggiaTreno (`http://www.viaggiatreno.it/`) websites, respectively for March and April 2015. The Swiss railway data is obtained from the OpenTransportData website (`https://opentransportdata.swiss/`) over January 2018. The delay data and network topologies are separate files, such that the topology is not inferred from the delay data files. No information on rolling stock and crew resources used in delay activities is known.

A.3 Infrastructure and service details of the Dutch railways.

The Dutch railways consist of 402 passenger stations, linked together in a dense network with the highest line utilisation rate and busiest tracks in Europe according to statistics from the European Union [3] and the Dutch Statistics Agency (CBS) [4]. Delay, location, time, train line number, activity nature, and more details are logged each time a train passes by a so-called 'service-control-point' (SCP) — which are 801 measure stations on the network, of which the passenger stations are a subset. The SCPs are used as nodes in this analysis, interlinked by 1438 edges (tracks between the SCPs). Network data on locations and links of the nodes and edges of the railway network, including delay time series can be found in an Open Science Framework related to previous work [1]: `https://osf.io/tps4r/`. The three layers contain, on average (December 2017, only NS), ∼6500 train service lines, ∼600 rolling stock units and ∼2300 crew members per day, respectively. The train lines are subdivided into 500 unique train *series* — groups of train lines that are relatively similar in their trajectories and type of rolling stock. ProRail and railway operators in the Netherlands classify each individual (whole) day as one out of four performance categories; 'Green', 'Neutral', 'Red' and 'Black'. The classification is based on train punctuality and cancellations — while Green days involve high punctuality and a low percentage of cancelled trains, Black days involve low punctuality and a high percentage of cancelled trains. The exact definitions are displayed in Tab. 1. Punctuality and cancellation percentages are calculated with a number of constraints, leading to an 'effective punctuality' and 'effective cancellation' percentage. First, only *arrival* activities are used. Secondly, only passenger trains are considered (no freight trains). Thirdly, only activities on a subset of stations are considered. These stations are chosen based on impact on not only future delay, but also passengers, pressure on personnel and infrastructure. This choice is the result of a joint concession with the Dutch Ministry of infrastructure [5]. The fourth and final constraint to calculate the effective punctuality and cancellation metrics is that these metrics are calculated on a few specified points on the day, and averaged. Note that the above is used to determine the classification of the days, while the results in this paper are not constrained by the above — in particular, our results are based on all stations.

Table 1. Day-to-day severity labels by Dutch asset manager ProRail. The last column shows the amount of days in the dataset (July 1st 2017 - June 30th 2018). The unofficial label 'Neutral' applies when none of the other labels does. Table re-used from [1].

**Label**	**Punctuality**	**Cancellations**	**Amount of days**
Green	$> 92.5\%$	$< 1\%$	46
Neutral	-	-	292
Red	$< 85\%$	$> 5\%$	21
Black	$< 75\%$	$> 10\%$	6

A.4 Crew rescheduling data

The data for on-the-fly mutations to the crew schedules have been obtained from Nederlandse Spoorwegen (NS). For every crew member, it contains not only the final, realized schedule, but also earlier stages of their schedules — with versions marked by an integer and accompanying *mutation time stamp* t_s. We need to use the latter to determine the version of the global crew schedule at any time: for example in the simulation of Fig. 3, or when calculating P in Fig. 5. To be precise, at any time t_0, all crew mutations with $t_s < t_0$ are known are used in the model starting at t_0. One difficulty we face for the mutation data is that every now and then, there are

system-wide updates of crew schedules: many mutations appear with identical time stamps as 'hindsight' updates at unrealistic times, e.g., at 02:00h on the day after. While these are clearly distinct from on-the-fly mutations carried out by traffic controllers, we cannot neglect them. In our simulations, we account for these by resetting the mutation times towards those at which the crew activities in question took place.

B Processing delay data

B.1 Delay calculation

As mentioned in the previous section, delays are discretely logged at the service-control-points, using indices to refer to certain activities: departure, arrival or passing-through. We define delay d of such an activity a as the difference between realised time $t_{\text{real}}(a))$ and the planned time $t_{\text{planned}}(a)$ of any activity:

$$d(a) = t_{\text{real}}(a) - t_{\text{planned}}(a) \tag{1}$$

This means that $d(a)$ is usually positive (when the train is late), but can be negative (when the train is early); the latter is rare due to the mandate that trains should not depart early. The transformation of discrete delay logs of individual activities to a continuous time series of delay may be useful for analysing the data itself (e.g. in principal component analysis as in previous literature [1]) or for visualization purposes. Note that this is not done in the model itself — in which we have discrete delay values for each activity (rather than a time series of delay in space). We therefore aggregate the discrete delay values per activity $d(a)$ to a time-dependent delay activity per edge e: $D(t, e)$. This is done by interpolating between the delay of departure $d(a_{\text{dep}})$ and arrival $d(a_{\text{arr}})$ activities of the same service, where the departure is being done on one side of edge e, and the arrival at the other side of e:

$$D(t,e) = \begin{cases} t - t_{\text{planned}}(a_{\text{dep}}) & \text{if } t_{\text{planned}}(a_{\text{dep}}) < t < t_{\text{real}}(a_{\text{dep}}) \\ d(a_{\text{dep}}) + \frac{d(a_{\text{arr}})-d(a_{\text{dep}})}{t_{\text{real}}(a_{\text{arr}})-t_{\text{real}}(a_{\text{dep}})} \cdot (t - t_{\text{real}}(a_{\text{dep}})) & \text{if } t_{\text{real}}(a_{\text{dep}}) < t < t_{\text{real}}(a_{\text{arr}}) \\ 0 & \text{otherwise} \end{cases} \tag{2}$$

Here, we use 1 second resolution, but in principle the aggregation can be done at any resolution. Depending on the purpose, Eq. (2) is done either per train, or aggregated over all trains that travel across the same edge at a particular time. The resulting time series are shapde in a sawtooth-like manner in which individual trains can be re-traced [6].

B.2 Delay jumps

A delay jump $\delta(a)$ is defined as the change in delay of activity a compared to its previous activity a_{previous}. Formally:

$$\delta(a) = d(a) - d(a_{\text{previous}}), \tag{3}$$

Note that if a is the train line's starting activity (i.e., there is no a_{previous}), then the delay jump is not defined. Activities a and a_{previous} can be of various types. For a delay jump at a station, a is the arrival of a certain train at the station, and a_{previous} its re-departure. Delay jumps at stations can arise due to, e.g., resource allocation problems and late transfers. Delay jumps can also occur on tracks, when a is the departure of a train onto a certain track, and a_{previous} is its arrival at the end of the

track. In Fig. 4 we consider *all* delay jumps of all possible types. In practise, railway companies often consider delay jumps to be only those values of δ that are larger than 3 minutes ('significant delay jumps'). We do not make this distinction and consider all values of δ to be 'delay jumps' — δ can even be negative, as can be seen in Fig. 4.

B.3 Classification of delay jumps

In Fig. 4, we label all delay jumps found in the data; while delay jumps can be found in the data, their causes are not recorded. Here, we describe each of these categories individually, using the (trilayer) model. The core of this exercise is that we compare the observed delay jump $\delta_{\text{obs}}(a)$ of every activity a with its simulated counterpart $\delta_{\text{sim}}(a)$ when initialising the model closely before the scheduled time of activity a, i.e. $t_0 < t_{\text{planned}}(a)$. In Fig. 4, model runs are reinitialised every 15 minutes, such that the maximum time between t_0 and $t_{\text{planned}}(a)$ is (less than) 15 minutes. Focusing on labelling delay jumps found in data, four categories can be found:

- I Cascading due to a delayed crew transfer. A delay jump is labeled (I) if $\delta_{\text{sim}} > 0$ because of a delayed crew member in the simulation output, and $\delta_{\text{obs}} > 0$.
- II Cascading due to a delayed rolling stock transfer. Analogous to label (I), delay jump is labeled (II) if $\delta_{\text{sim}} > 0$ because of a delayed rolling stock unit in the simulation output, and $\delta_{\text{obs}} > 0$.
- III Delay jumps larger than 10 minutes, but unaccounted for in the model.
- IV Noise, i.e. all other delay jumps. Individual units in this category, as well as the net sum within a time window can be both positive and negative (as seen in Fig. 4.

However, when for any train activity a, a (nonzero and positive) delay jump is found in the model output, but not in the observed data, we distinguish two reasons describing this discrepancy:

- V Mitigation of the delay jump (δ_{sim}) due to rescheduling of the activity: if the simulation initialised closest to $t_{\text{planned}}(a)$ did not predict a positive delay jump, but other simulations (initialised earlier) did, we assume that changes in the schedules were made to mitigate this delay jump.
- VI Mitigation of the delay jump (δ_{sim}) due to cancellation of the activity. Whether a train activity is cancelled can be found directly in the raw data, so no heuristic is needed here.

To calculate the magnitude of mitigation labels (V and VI), we use the simulated delay jumps δ_{sim} rather than the observed ones δ_{obs} as used in labels I-IV. In Fig. 4, all delay jumps (δ_{obs} for labels I-IV, δ_{sim} for labels V-VI) are accumulated per label within a certain time window. The relative importance $\nu(l)$ of each label l can then be calculated as:

$$\nu(l) = \frac{\sum_{a|\delta(a) \text{ has label } l} \delta(a)}{\sum_a \delta(a)} \tag{4}$$

where a denotes train activities.

B.4 Plotting details

For plotting delay data in Figs. 1 and 3, a smoothening procedure is applied to the spatial representation of delay, by applying a weighted averaging of neighbouring edges delay values. The weights are 1 for the edge considered, and 0.25 for neighbours. This is only done for visualisation purposes, and analyses include only raw values. In Fig. 4, a

running window of 5-minutes length (i.e., +/- 2.5 minutes) is used at each (1 minute-) time step, followed by a 30-minute running mean smoothening over all the percentage curves. In Fig. 4b, four days per class are used from the period 1 July 2017 to 30 June 2018. In particular, these included 11 Dec. 2017, 23 Nov. 2017, 1 May 2018 and 30 Apr. 2018 for Black days; 18 Nov. 2017, 20 Nov. 2017, 22 Nov. 2017 and 13 Dec. 2017 for Red days; 21 Nov. 2017, 3 Dec. 2017, 28 Nov. 2017 and 16 Dec. 2017 for Neutral days; and 28 July 2017, 15 Apr. 2018, 22 July 2017 and 17 Sep. 2017 for Green days. The days were selected randomly in the dataset. To calculate the averages in Fig. 4b, only time windows around points between 6:00 AM and 1:00 AM (next day) are taken, to avoid confusing biases in the middle of the night (when only very few trains are travelling).

C Model details

C.1 Model basics

The basic principle of the mono-, bi- and trilayer models used in this work is the explicit simulation of the planned schedules. Starting the the model at any chosen point in time t_0, it iterates over all subsequent planned activities $a \in \{a(t)|t > t_0\}$, sorted by scheduled activity time (a list of roughly 150,000 activities if initialising at the start of day) — that is, when an activity $a(t_i)$ is concerned, the delay of all activities before that ($\{a(t)|t < t_i\}$) are already determined. For each iteration (activity), the delay is calculated and attributed if any of the following conditions are met:

- The train line involved in a had delay in the previous activity (sometimes referred to as 'advection').
- A rolling stock unit used in this activity was transferred from another train line, and had delay.
- A crew unit used in this activity was transferred from another train line, and had delay.

This is captured in Eq. (1) in the main text. Although possible, in this paper we did not include any noise, such that delay passed on without any (stochastic) reduction (see below under 'Noise'), but buffers are always taken into account.

C.2 Formal model

Given the set of all train service activities $\{a(t)\}$ with t referring to their planned time, we initialise the model at time t_0 with knowledge about all activities $\{a(t)|t \leq t_0\}$. The aim of the model is to simulate how delay spreads towards other activities later than t_0: $\{a(t)|t > t_0\}$. In the monolayer model, we propagate delay of activity $a_0 \in \{a(t)|t \leq t_0\}$ to future activity $a_1 \in \{a(t)|t > t_0\}$ if a_1 is the subsequent activity of the train service performing a_0, minus a scheduled buffer $\beta(a_0, a_1) = t_{\text{plan}}(a_1) - t_{\text{plan}}(a_0)$ for each activity i. Similarly, in the bilayer model, we not only propagate delay from a_0 to a_1 when they are performed by the same train service, but also when the same rolling stock unit is used. In the trilayer model, delay is propagated via also the reusage of crew members. In general, we can express the potential delay propagation that may be propagated from activity a' to subsequent activity a as $\mathcal{H}[d(a') - \beta(a', a)]$. The Heaviside function $\mathcal{H}(x)$ is used to prevent trains from departing early (which in practise rarely happens, and if so, with small delay jumps). For any activity $a \in \{a(t)|t > t_0\}$, then, we compute its delay from delays of previous activities $a' \in \{a(t)|t \leq t_0\}$ as in Eqn. (1), using the maximum of all potential propagations of delay — of all unique resources used in activity a.

C.2.1 Buffers

Concerning the matter of buffers, we include up to 5 minutes as a necessary transfer time for resources. For example, if a crew member has 14 minutes to transfer towards another train, we assume that it needs at least 5 minutes to get to that train, leaving an effective buffer of only 9 minutes (which is then used in the calculation). If the planned buffer would be 3 minutes, this results in 0 minutes buffer — instead of a negative buffer of -2 minutes, we assume that a 3-minute planned buffer must be associated to an easy transfer that does not require 5 minutes. The role of buffers in the in delay propagation has been investigated in-depth previously [7], but it is not the goal of this paper.

C.3 Initialisation.

Initialising the model from a real snapshot in the past (e.g., as in Fig. 3) requires extra attention. Trivially, the train activities that were realised before the initialisation time (19:00) need to be put in by the data (as 'initialisation'), and those far past 19:00 need to be fully calculated by the model. However, the simulation of delays of train activities realised just after 19:00 are less trivial — as some information is already available about these delays at 19:00 meaning that the delay can be more accurately estimated. We treat these boundary cases in the following manner (assuming positive delay):

- If $t_{\text{plan}}(a_{\text{dep}}) < t < t_{\text{real}}(a_{\text{dep}})$: only $d(a_{\text{dep}})$ is initialised from the data, and $d(a_{\text{arr}})$ is extrapolated using only real-time information: equal to $d(a_{\text{dep}})$.
- If $t_{\text{real}}(a_{\text{dep}}) < t < t_{\text{real}}(a_{\text{arr}})$: both $d(a_{\text{dep}})$ and $d(a_{\text{arr}})$ are initialised from the data.

Negative delays in a similar manner, but the case of $t_{\text{real}}(a_{\text{dep}}) < t < t_{\text{plan}}(a_{\text{dep}})$ arises, where also only $d(a_{\text{dep}})$ is initialised from the data.

C.4 Noise

As shown in Eq. (1), noise can be put in the model to simulate small (new) delay jumps, as in the real data: delay changes due to numerous small events like slow driving due to difficult weather conditions or a large number of passengers being slow to get into the train. For the model results in this paper, we set noise ζ to zero, in order to avoid confusion between model noise and noise in the real data; inclusion of model noise would have made interpretation of delay jumps in Fig. 4 much more difficult.

In the past, we have used a variation of this model that included explicit noise, by estimating noise from the data of all delay jumps per aggregated edge e and train series s over 100 days [1] — e.g., by considering all delay jumps of the 3000 train service series between Utrecht and Amsterdam. From this, we assembled a probability density function (PDF) per such pair (e, s). In that earlier work, for calculating the delay of activity a as in Eq. (1), we drew the (uncorrelated) noise from these PDFs. As we ran a similar procedure for this paper; we found that cascading effects are much stronger than the effect of noise for large-scale disruptions, leading us to the conclusion that stochasticity does not play a major role in their development. For this reason, as well as to improve interpretability of the real data as in fig. 4, we have excluded explicit noise from the model.

C.5 Mitigation elements

The model describes delay propagation, given that the schedules of train lines and assets remain the same, i.e., *if dispatchers take no mitigation actions* (although we do quantify mitigation in hindsight in Fig. 4). This assumption reduces the model's

predictability horizon. In practise, the Dutch railway organisations have various more detailed contingency plans (*Versperringsmaatregelen*) in place, partially predetermining action in given disruption events. These involve protocols on highly specific situations which are beyond the scope of this research, but may in the future be used to predict delay development even further (e.g., specifically for the Dutch railways). For the purpose of model performance metrics, we exclude trains that end up being cancelled (as we have no prediction means for that in the model). Mutations on the crew schedules are not implemented throughout a single model simulation, but re-initialising multiple runs with different version of the schedules can be utilized to have updated information on what happens in the real data (see Figs. 4 and 5).

References

1. Dekker MM, Panja D, Dijkstra HA, Dekker SC. Predicting transitions across macroscopic states for railway systems. PLOS ONE. 2019;14(6):e0217710. doi:10.1371/journal.pone.0217710.

2. Monechi B, Gravino P, Di Clemente R, Servedio VDP. Complex delay dynamics on railway networks from universal laws to realistic modelling. EPJ Data Science. 2018;7(1):35. doi:10.1140/epjds/s13688-018-0160-x.

3. European Commission. Statistical Pocketbook EU Transport. European Union. 2016;.

4. Ramaekers P, Wit Td, Pouwels M. Hoe druk is het nu werkelijk op het Nederlandse spoor? Centraal Bureau voor de Statistiek. 2016;.

5. Concessie voor het hoofdrailnet 2015-2025. Ministerie van Infrastructuur en Milieu. 2014;.

6. Dekker MM, Panja D. A reduced phase-space approach to analyse railway dynamics. IFAC-PapersOnLine. 2019;52(3):1–6. doi:10.1016/J.IFACOL.2019.06.001.

7. Zieger S, Weik N, Nießen N. The influence of buffer time distributions in delay propagation modelling of railway networks. Journal of Rail Transport Planning and Management. 2018;8(3-4):220–232. doi:10.1016/j.jrtpm.2018.09.001.
